# Reliability in adolescent fMRI within two years – a comparison of three tasks

**DOI:** 10.1038/s41598-017-02334-7

**Published:** 2017-05-23

**Authors:** Nora C. Vetter, Julius Steding, Sarah Jurk, Stephan Ripke, Eva Mennigen, Michael N. Smolka

**Affiliations:** 1 Department of Psychiatry and Neuroimaging Center, Technische Universität Dresden, Dresden, Germany; 2 Department of Child and Adolescent Psychiatry, Faculty of Medicine of the TU Dresden, Dresden, Germany; 3 Department of Psychology, Bergische Universität Wuppertal, Wuppertal, Germany; 4 Division of Psychological and Social Medicine and Developmental Neurosciences, Faculty of Medicine of the TU Dresden, Dresden, Germany

## Abstract

Longitudinal developmental fMRI studies just recently began to focus on within-subject reliability using the intraclass coefficient (ICC). It remains largely unclear which degree of reliability can be achieved in developmental studies and whether this depends on the type of task used. Therefore, we aimed to systematically investigate the reliability of three well-classified tasks: an emotional attention, a cognitive control, and an intertemporal choice paradigm. We hypothesized to find higher reliability in the cognitive task than in the emotional or reward-related task. 104 healthy mid-adolescents were scanned at age 14 and again at age 16 within M = 1.8 years using the same paradigms, scanner, and scanning protocols. Overall, we found both variability and stability (i.e. poor to excellent ICCs) depending largely on the region of interest (ROI) and task. Contrary to our hypothesis, whole brain reliability was fair for the cognitive control task but good for the emotional attention and intertemporal choice task. Subcortical ROIs (ventral striatum, amygdala) resulted in lower ICCs than visual ROIs. Current results add to the yet sparse overall ICC literature in both developing samples and adults. This study shows that analyses of stability, i.e. reliability, are helpful benchmarks for longitudinal studies and their implications for adolescent development.

## Introduction

To date, the field of longitudinal developmental fMRI studies is growing^1^. However, it remains largely unclear which degree of quantitative reliability can be achieved in developmental studies.

The preferable quantitative reliability measure in fMRI studies is the intraclass coefficient (ICC^2^) with the following formula:

1 $${\rm{ICC}}(3,1)=\frac{M{S}_{between}-\,M{S}_{error}}{M{S}_{between}+(k-1)M{S}_{error}}$$

The total sum of squares in this model is split into between-subjects (MS_between_) and error (MS_error_) mean sums of squares and k represents the number of obvervations^3^. The ICC ranging from 0 to 1 tells us how much variance from the total variance in two measurements is due to variance between participants. An ICC of 1 would imply that participants’ brain activation does not change over time (no within-subject variance). ICCs are classified according to Cicchetti^4^ as poor (<0.40), fair (0.41–0.60), good (0.61–0.75), and excellent (>0.75)^5^. So far, almost exclusively adult neuroimaging studies measured reliability and found large variance across studies with an average ICC of 0.5^2^. According to Cicchetti^4^, this ICC can be classified as ‘fair’. These methodical studies measured only small samples of 10 to 20 adults in a short time span from a few days to a few weeks^2^.

However, it remains largely unanswered if these test-retest reliabilities can be generalized to typical developmental longitudinal samples, which usually span larger time intervals between measurements. There have been only two previous developmental studies that reported ICCs^6, 7^. Van den Bulk *et al.* ^7^ investigated n = 20 12 to 19 year-old adolescents and obtained fair reliability for the prefrontal cortex (PFC) and poor reliability for the amygdala using an emotional faces task. Koolschijn *et al*.^6^ used a cognitive rule-switch task and showed fair to good reliability for n = 12 15 year-old adolescents. The two studies of van den Bulk *et al*.^7^ and Koolschijn *et al*.^6^ differ in their investigated age-range, time interval (van den Bulk *et al*. 3 months; Koolschijn *et al*. 4 years) and their employed task domain that was either cognitive or emotional. Thus, evidence on reliability in developmental studies remains sparse. To fill this research gap the current study aimed at analyzing reliability in a large sample of 104 14-year old adolescents measured within a time interval of 2 years. Methodically, we focused on two important factors that can influence reliability: the task domain and the region of interest (ROI).

The task domain is a first factor that might influence fMRI reliability. Adult studies showed that reliabilities differed between task domains such as cognitive, emotional, or reward-related^2^. Only one adult study compared the reliability between these task domains using specific ROIs in one sample^8^. Results indicated a poor ICC for the amygdala in an emotional faces task, fair ICCs for frontal and parietal regions in a cognitive N-Back task, and fair to good ICCs in the ventral striatum (VS) for a reward task. Taken together, this study suggests that ICCs might be higher in cognitive and reward-related compared to emotional task domains.

Currently, there is no developmental reliability study comparing task domains. This is surprising since a recent review on developmental longitudinal studies suggests emotional and reward-related tasks might show lower test-retest reliability than cognitive tasks^1^. This was concluded from findings of low reliability, both for amygdala activity in emotional tasks^7, 9, 10^ and VS activity in reward tasks^11, 12^. In contrast, the prefrontal and parietal cortex showed relative high reliability in cognitive control tasks^6, 13^. Most of these studies except Koolschijn *et al*.^6^ and van den Bulk *et al*.^7^, however, did not measure adolescent ICCs but either analyzed only Pearson’s correlations of time point one and two^11, 12^, only reported on group differences of activation from time point one and two^9, 10^, or analyzed ICCs only in an adult sub-sample^13^. In contrast to Pearson’s correlations the ICC provides a more accurate estimate because it can distinguish between systematic variation and average consistency over time^14^. Group differences are also not appropriate for conclusions about reliability because they only compare activation on a group level instead of an individual level. Therefore, the ICC is most suited as a quantitative intra-individual measure of reliability.

With this in mind, for the first time, we aimed at systematically comparing an emotional, a cognitive, and a reward-related task in an adolescent sample. The emotional task has been shown to yield valid results both on the behavioral and neural level^15, 16^. It activates the fusiform gyrus, the inferior and middle frontal gyrus, and the inferior parietal lobe. Amygdala activation for negative stimuli in this task has been demonstrated to be sensitive towards a family history of depression in healthy adolescents^15^. The cognitive control task has shown robust switch and interference effects on the behavioral and on the neural level^17, 18^. Further, the neural overlap between the switch and interference effect has revealed brain activation in the dorsal anterior cingulate cortex (dACC), the dorsolateral prefrontal cortex (dlPFC) as well as the posterior parietal cortex (PPC)^17^. The intertemporal choice task^19^ is a widely used task that activates the VS for value processing and the ACC, PFC, and PPC for intertemporal decision making^20–22^. Developmental change in activation from age 14 to 16 has only been found for the emotional attention task^16^, while the other tasks did not yield developmental effects^23, 24^.

A second factor influencing fMRI reliability is the chosen ROI. While developmental emotional tasks suggest lower reliability for the amygdala^1, 7^, higher reliabilities seem to result for occipital regions^7^. Previous studies mostly focused on only one or two regions such as the amygdala for emotional tasks^8, 25, 26^. Here, we analyzed three to five functional ROIs important for the respective task to achieve an overall picture of test-retest reliability. Additionally, we analyzed the whole brain ICC because it calculates the global concordance of neural activation regarding all voxels and therefore has been suggested to be the strictest criterion of fMRI reliability^2^.

While considering the two important factors task domain and ROI, other parameters that might influence reliability^2, 8^ were held constant: scanner, scanning parameters, sample size, time interval, and event-related task design across all paradigms. We expected that the task of the cognitive domain would show higher reliability than that of the emotional or reward-related domain considering adult^8^ and current developmental literature^1^.

## Results

### Behavioral reliability

Behavioral reliability was fair in all behavioral measures of the three paradigms except for the overall reaction time of the cognitive control task, in which reliability was good (see Table 1). This fair to good reliability fits to the behavioral developmental effects in all paradigms: Adolescents became faster from age 14 to 16 in both the emotional attention^16^ and cognitive control paradigm. The log-transformed discount parameter increased which can probably be interpreted with decreased impulsivity from age 14 to 16^22^.

**Table 1:** Behavioral data at both time points and resulting ICCs.

Task	Behavioral Measure	T1 - ms	T2 - ms	t/p^a^	d^b^	ICC_(3,1)_
		M (SD)	M (SD)			(95%-CI)
Emotional attention	RT (overall)	719 (85)	696 (101)	2.33/0.022	0.24	0.46 (0.29–0.60)
	RT (negative attended)	726 (87)	700 (109)	2.45/0.016	0.26	0.42 (0.25–0.57)
Cognitive control	RT (overall)	906 (151)	826 (127)	7.15/<0.001	0.57	0.67 (0.55–0.76)
	RT (switch incongruent)	992 (162)	905 (142)	7.04/<0.001	0.57	0.46 (0.29–0.60)
Intertemporal choice	log_k^c^	−4.73 (0.79)	−4.93 (0.98)	2.18/0.032	0.22	0.47 (0.31–0.61)

Note: ^a^t-test for paired samples comparing T1 and T2 values; ^b^Cohen’s d for the standardized mean difference; ^c^log-transformed discount parameter, for methods, see Ripke *et al*.^22^.

### FMRI reliability

#### Whole brain ICCs

**Figure 1:**
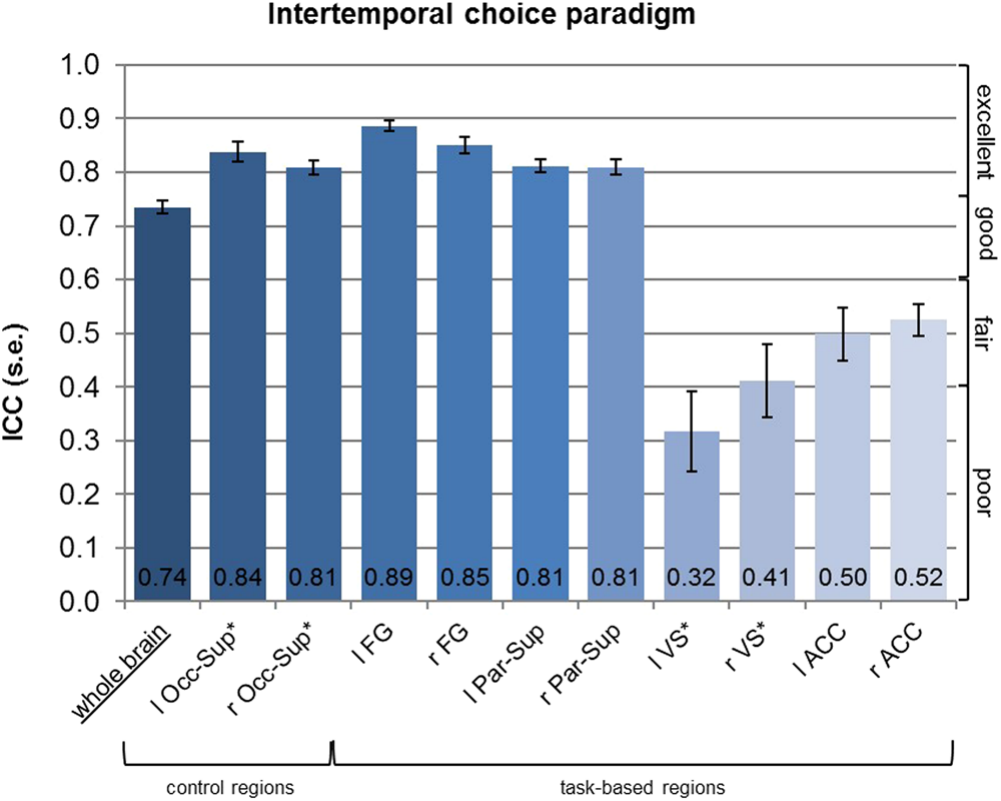
Results of ICC analyses for the intertemporal choice paradigm: *These regions are based on anatomical masks (AAL). l – left, r – right, Occ-Sup – Superior occipital lobe, FG – Fusiform gyrus, ACC – Anterior cingulate cortex, Par-Sup – Superior parietal lobe.

**Figure 2:**
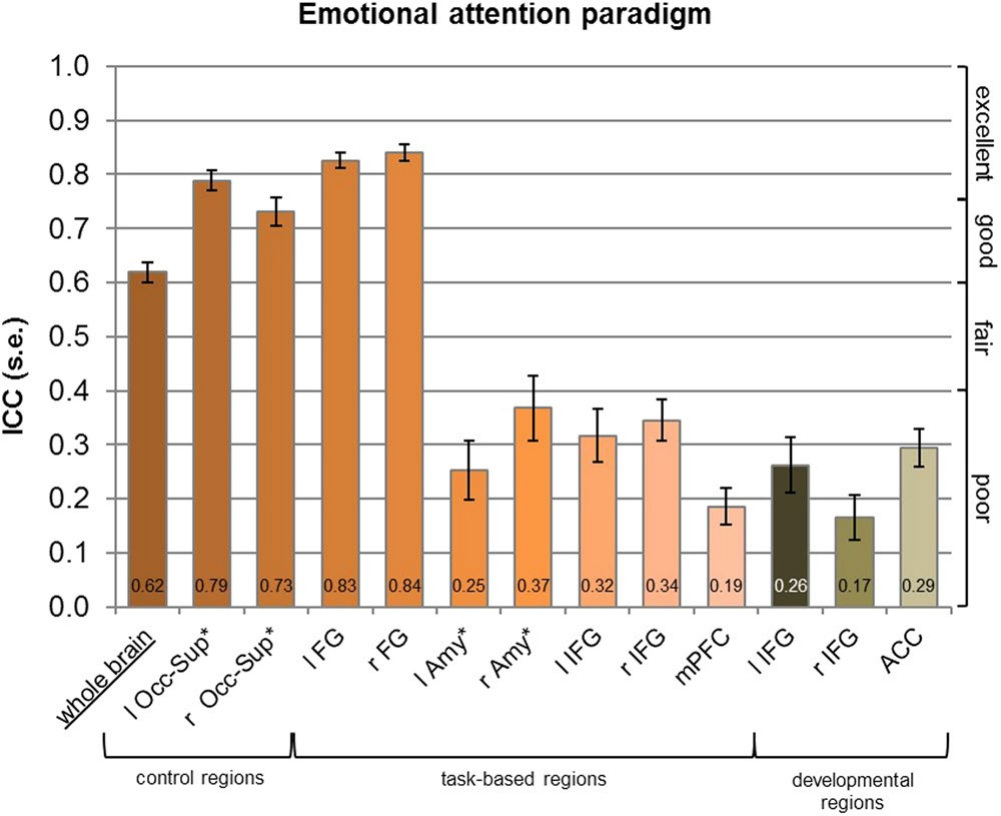
Results of ICC analyses for the emotional paradigm. *These regions are based on anatomical masks (AAL). l – left, r – right, Occ-Sup – Superior occipital lobe, FG – Fusiform gyrus, Amy –Amygdala, IFG – Inferior frontal gyrus, mPFC – Medial prefrontal cortex, ACC – Anterior cingulate cortex.

**Figure 3:**
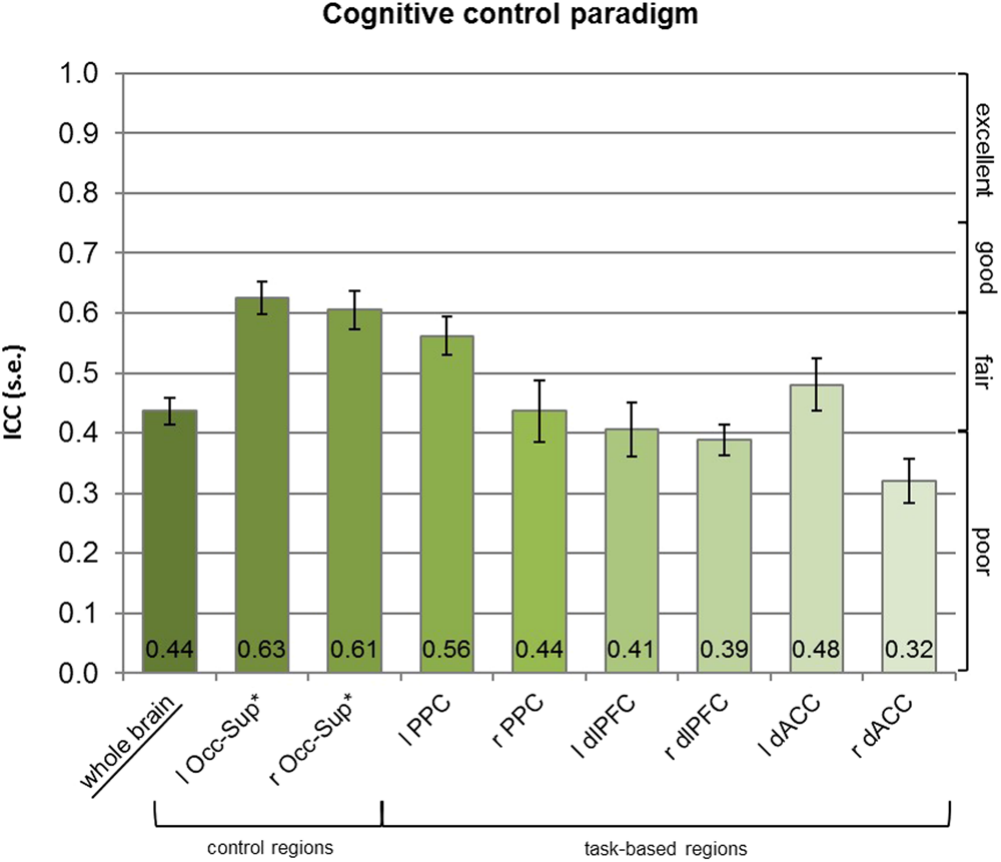
Results of ICC analyses for the cognitive control paradigm. *These regions are based on anatomical masks (AAL). l – left, r – right, Occ-Sup – Superior occipital lobe, PPC – Posterior parietal cortex, dlPFC – Dorsolateral prefrontal cortex, dACC – Dorsal anterior cingulate cortex.

The whole brain ICC of the reward paradigm was highest across paradigms, 0.74 (see Fig. 1), and together with the emotional attention paradigm, 0.62 (see Fig. 2), it was in the “good” range. The ICC of the cognitive control paradigm was lower and only in the fair range, 0.44 (see Fig. 3). An ANOVA showed that the whole brain reliability differed significantly between the paradigms (*F* = 102.67, *p* < 0.001, η^2^_partial_ = 0.499) with post-hoc analyses revealing that whole brain reliability of the reward paradigm was higher than emotional attention, which was higher than cognitive control (with all paradigms differing significantly from another, *p*’s < 0.001).

#### ICCs of different ROIs

For the task-based ROIs in the emotional attention paradigm, ICCs were poor (amygdala, IFG, medial prefrontal cortex, mPFC) to excellent (fusiform gyrus, FG) ranging from 0.19 to 0.84 and poor for the development-based ROIs (ACC, IFG) ranging from 0.17 to 0.29 (see Fig. 2). A Wilcoxon signed-rank test revealed that the task-based IFG ROI was higher than the development-based IFG ROI (*p* = 0.002 for the left IFG and *p* = 0.001 for the right IFG). For the cognitive control paradigm, ICCs ranged from 0.32 to 0.56. ICCs were thus poor to fair for the dlPFC and dACC and fair for the PPC (see Fig. 3). The intertemporal choice paradigm yielded poor to fair ICCs for the VS and ACC ranging from 0.32 to 0.52 and excellent ICCs for the superior parietal lobe (Par-Sup) and the FG ranging from 0.81 to 0.89 (see Fig. 1). The control region in the occipital cortex (superior occipital lobe, Occ-Sup) yielded good to excellent reliability across paradigms.

## Discussion

The current study aimed at investigating reliability in a large sample of mid-adolescents in three important domains of information processing using an emotional, a cognitive, and a reward-related task. We also considered different ROIs while holding other parameters that might influence reliability constant. Results showed that behavioral reliability was fair for all three paradigms. For fMRI reliability, the cognitive task yielded only fair whole brain reliability whereas the emotional and the reward-related task showed good whole brain reliability. ICCs of ROIs depended largely on the specific regions and the task and ranged from poor to excellent. Overall, ICCs were comparable to previous adult studies^2^.

In terms of behavioral reliability from age 14 to 16, we found fair to good ICCs. For the emotional and the cognitive task, the participants became faster, while the reward-related task indicated less impulsive behavior, which is in line with previous studies^27–29^. Low behavioral reliability can be expected for tasks with developmental changes.

For our first aim, to explore whether functional imaging reliability depends on the task domain, the whole brain ICC was chosen. This ICC has been suggested to be the strictest approach for reliability^2^, because it assumes on a whole-brain basis that the level of activity in all voxels should remain constant irrespective of suprathreshold activation. All three paradigms obtained a fair to good whole brain reliability. The reward paradigm had the highest whole brain ICC of 0.74 which can be classified as good to excellent. The emotional task had a whole brain ICC of 0.62 that was significantly lower but still in the good range. The ICC of the cognitive task differed significantly and was only in the fair range (ICC = 0.44). Thus, our first hypothesis, that the cognitive task would show higher reliability than the emotional or reward-related task (see also ref. 1) was not supported. To the contrary, the reward-related task yielded highest reliability followed by the emotional and the cognitive task. Our findings do not support the conclusions of Plichta *et al*.^8^, probably because they measured adults and investigated the amygdala only for their emotional task while we also investigated whole brain ICCs. Speculatively for the cognitive paradigm, the low behavioral reliability might probably be related to the low fMRI reliability. However, when exploring correlations of behavioral and fMRI ICCs we did not find such a relationship (see S4 in the supplements). Furthermore, the lower whole brain ICCs of the cognitive control paradigm could stem from lower ICCs in the occipital regions (0.61 and 0.61 as opposed to 0.79, 0.73, 0.84 and 0.81 for the other two paradigms), respectively higher ICCs in the emotional attention paradigm for lower processing regions such as the IFG. The conclusion regarding lower reliability in the cognitive control paradigm has thus to be taken cautiously and investigated further in future studies.

Regarding our second aim, the single analyzed ROIs, the control region in the occipital cortex yielded good to excellent reliability across paradigms. The high reliability for the occipital cortex in the emotional paradigm is in line with another adolescent study^7^. The rather low-level visual area fusiform gyrus also yielded excellent reliability in both the emotional and reward task in line with previous emotional adult studies^30, 31^. In contrast, other regions that are relevant for cognitive or emotional-motivational processes such as subcortical (amygdala, VS) and cortical regions (PFC) showed low reliability. Taken together, the current study suggests that across three tasks in the same sample reliabilities might be higher in regions of basic visual processing compared to cognitive or emotional-motivational brain regions. This might be due to higher variability in higher-level cognitive processes than basic visual processing^32^. Another explanation might be that developmentally, visual regions have already matured, while subcortical and cortical higher-level regions continue to develop in adolescence^33, 34^.

In the following the regions that are relevant for cognitive or emotional-motivational processes are discussed for each paradigm separately.

For the *emotional attention paradigm* we found poor amygdala ICCs. Only one previous study investigated *adolescent* amygdala reliability with an age-heterogeneous sample of n = 20 12 to 19 year-olds^7^ and found poor reliability within a short interval of 3 months. Our results show that poor amygdala reliability is also evident in a large sample of mid-adolescents within a longer time interval of 2 years.

From a developmental perspective, current results can be integrated with previous findings of a potential peak in amygdala activation in mid-adolescence compared to child- and adulthood (for a review, see refs 1 and 33). While some previous cross-sectional studies have supported this amygdala peak^35, 36^, longitudinal studies rather indicated “relative stability” in amygdala activation across mid-adolescence^9, 16^. The current sample is a sub-sample of our previous longitudinal study that did not find amygdala activation change from age 14 to 16^16^. Therefore, current results suggest that this “relative stability” and lack of peak in mid-adolescence might occur at the same time as intra-individual variability, i.e. low reliability in amygdala activation (in accordance with the conclusions of a recent review)^1^.

It is also possible that the amygdala signal itself might be instable, independent of development^1^. This is supported by adult studies that also found poor to fair amygdala ICCs in emotional tasks^8, 25, 26, 30, 31^.

Regarding frontal regions important for emotional processing^16^, the first region IFG showed poor reliability in line with a previous emotional adult study^31^. An emotional adolescent study found that IFG activation at baseline correlated with activation 2 years later indicating some degree of reliability^37^. The second region, mPFC, showed poor reliability similar to the adolescent study of van den Bulk *et al*.^7^. In our previous longitudinal study^16^, part of the IFG and the ACC demonstrated a developmental effect, i.e. higher activation at age 16 than 14. Expectedly, this developmental region showed a lower reliability than the (larger) IFG ROI that was functionally defined at age 14. The ACC showed a poor reliability similar to an adult study^31^.

The *cognitive control paradigm* showed poor to fair ICCs partly in contrast to the only other adolescent study^6^ that found good ICCs for the PPC and dACC while the dlPFC result was in a similar fair range. But it should be noted, that the ACC of Koolschjin *et al*.^6^ was located more anteriorly. Also an adult study found good to excellent ICCs^38^. However, there are not many studies that have calculated ICCs in cognitive control tasks. Cognitive control can be divided into three related factors: inhibition, shifting, and updating^39^. The current interference *and* switch task assesses both inhibition and shifting. No previous study examined ICCs using such a task. Taking updating tasks into account, current results are in line with ICC ranges of adult studies (Plichta *et al*.^8^ using an n-back task, Brandt *et al*.^40^ using a memory encoding task, and Bennett and Miller^41^ using an episodic and two-back memory task). We speculate that ICCs in our task may be low, as it assesses two cognitive control functions simultaneously. Unfortunately, due to our task design it is not possible to separate both components of cognitive control (i.e. task switching and overcoming incongruence) because each trial contains information on incongruence as well as task switching. Future studies should systematically compare ICCs of more basic cognitive control tasks.

To our knowledge this is the first study that tested reliability of a *reward-related paradigm* in an adolescent sample. The intertemporal choice paradigm showed fair to good ICCs in the superior parietal lobe and the ACC, which is in line with previous adult studies (probabilistic reversal task^42^; classification learning task^43^). For the VS, our results were in the poor to fair range, which is in line with Chase and colleagues^44^ using a card guessing task re-scanned within one week. In contrast, Plichta *et al*.^8^ found excellent ICCs in the VS for a reward task within two weeks. Our findings of low VS reliability are in line with the conclusions by Crone & Elzinga^1^ that there might be large variability in subcortical brain regions (amygdala, VS) in adolescence.

The reliability of fMRI data has implications for longitudinal studies of reward processing, which are pivotal to detect developmental change in brain-behavior relations. For example, Braams *et al*.^45^ assessed response to rewards in participants aged 8 to 25 longitudinally within 2 years and found an inverted U-shaped activation of the VS with a peak in activation during adolescence. This peak was also found behaviorally in a balloon analog risk taking task. A further longitudinal study was able to extent knowledge about dynamics of reward anticipation on the brain and behavioral level in adolescents^11^. Results showed that changes in VS activation over 2 years were related to changes in the behavioral approach system fun seeking score^46^ during the same time period. A third longitudinal study found increasing dorsal striatal activation from mid-adolescence to late-adolescence/early adulthood in response to anticipation of gain and loss^12^. Taken together, reliability of reward-related activation seems to depend on time between measurements and brain regions. While ICCs of cortical areas were mostly good to excellent, the results regarding the subcortical area VS are not conclusive. Additionally, ICCs have to be interpreted with respect to expected developmental-related changes regarding activation patterns. Thus, additional studies are needed to systematically investigate this relationship.

Overall, current results warrant discussion with regard to the following considerations and limitations. The ICC depends on the between-subject variance. Thus, current results might be related to the type of the current sample that is rather homogenous (fine-grained age range, similar sociodemography, intelligence, and pubertal status). Future studies could test reliability using more heterogenous samples.

Similar to other adolescent reliability studies^6^ this study was not designed a priori as a methodological study that investigates reliability but part of an overall research project focusing on adolescent brain development in several domains. The large sample size spanning about 200 participants (before exclusion due to movement, technical or behavioral outliers, see S1 in the supplement) required a time span of about 2 years. Because of this time span and the developmental sample we can therefore not disentangle between reliability due to development or reliability which would have occurred without development (e.g. in an adult population).

Assuming that changes in brain processes will be more likely to occur in contrasts which are expected to be effected by development (i.e. specific contrasts, like decision for small immediate vs. larger later in the intertemporal choice task), we used more general contrasts to investigate the reliability of the imaging data in our large sample. Although reliability and developmental changes are not two sides of the same coin, both are harder to distinguish the more developmentally sensitive the contrast is. Therefore, our rational was that, if the reliability of the more general contrasts would be moderate to high, the imaging data per se might be reliable; in the current study even over a timespan of two years.

As this area is still controversial, we chose baseline contrasts after careful consideration, since their constancy allowed us to compare single conditions of different paradigms more clearly as opposed to two contrasted conditions per paradigm. Especially in the developmental literature, the importance of differentiating between baseline and higher level contrasts has been emphasized^1, 47^ to infer more precisely which contrast led to developmental effects: in case of developmental changes in a higher level contrast, it is not possible to conclude what has changed: condition A, condition B, or both^1, 47^. Furthermore, it has been suggested that baseline contrasts yield better reliability than higher level contrasts^8^. However, current results have to be considered carefully and with potentially lower ICCs for higher level contrasts in mind.

Nevertheless, the study is unique due to its large sample and the three tasks that were tested for reliability. Future studies could systematically assess reliability in a (smaller) adolescent sample within a short time span and at the same time systematically control for potential changes in several domains (development, cognitive strategy, motivation etc.) and compare tasks that show developmental change in adolescence and those which do not. The reliabilities could further be compared to an additional adult population.

This study contained a qualitative comparison between tasks and was not designed a priori to systematically compare reliabilities of parallelized tasks. There were several aspects that could not be controlled for in the current analyses. First, the number of specific trials for the chosen contrast differed between tasks. While the task with the highest amount of trials was the most reliable one, the emotional attention task had fewer trials than the cognitive control task but a higher reliability, which might not fit to the conclusion that amount of trials correlates with task reliability. Second, behavioral differences that might stem from changes in performance, cognitive strategy or task focus^48–50^ could not be controlled for. Third, the implicit baseline that was included in all regressors of interest differed between tasks (length of fixation cross and cognitive process during baseline). Fourth, due to each paradigm’s specific effect size functional ROIs were created specifically for each paradigm: the statistical thresholds for the second-level analyses that built the basis of the definition of the functional ROIs differed between paradigms as well as the approach to rely on the peak voxels (emotional attention, cognitive control) or the anatomical overlap (intertemporal choice). Future studies should hold these features between tasks constant or control for them to be able to systematically compare task domains without potential confounders. ROIs were defined on the group level instead of the individual level similar to other studies^20, 51–55^. Future studies could also add ROIs based on the individual level and calculate reliability.

Taken together, ICCs in each paradigm were largely dependent on the respective ROIs with subcortical ROIs (VS, amygdala) resulting in lower ICCs than visual ROIs. The emotional and reward paradigm had higher whole brain ICCs than the cognitive paradigm. Current results add to the yet sparse overall ICC literature in both developing samples and adults. In the different task domains, ICCs were similar as in adult studies. To test whether results are specific for adolescents or can be generalized to adults the current paradigms could be tested in adults. Analyses of stability, i.e. reliability, are helpful benchmarks for longitudinal studies and their implications for adolescent development.

## Material and Methods

### Participants

The institutional review board of the medical faculty of the TU Dresden approved the study and the study was realized in accordance with it and with the Declaration of Helsinki. Participants were recruited from local schools and received monetary compensation for their participation. Written informed consent was obtained from both the participants and one of their legal guardians. The current dataset stems from the overall project “The adolescent brain”^22^ that investigated 250 adolescents at age 14 and again at age 16. For technical and practical issues not all of these participants completed all three tasks at both time points.

Sub-populations of this sample were previously reported regarding cross-sectional analyses of age 14 (emotional attention task, n = 164, Pilhatsch *et al*.^15^, intertemporal choice task, n = 235, Ripke *et al*.^22^; n = 206, Ripke *et al*.^56^, cognitive control task, n = 184, Mennigen *et al*.^17^, Rodehacke *et al*.^18^) or longitudinal change from age 14 to 16 (emotional attention task, n = 144, Vetter *et al*.^16^, intertemporal choice task, n = 80, Ripke *et al*.^23^). We here report on the overlapping sample of 104 healthy participants who performed all three tasks at age 14 and 16 successfully. This sample was analyzed for reliability for the first time.

For information of exclusion criteria for each task see Supplement S1. Participants had normal or corrected to normal vision and neither any record nor any current diagnoses of neurological, psychiatric, or serious medical disorders. Current psychiatric disorders were identified with the Development and Well-Being Assessment (DAWBA^57^). General cognitive ability of the sample was in the average to above average range (IQ across both time points: *M* = 115; *SD* = 10; range = 89–139) and did not change between measurements (*t* = 1.03; *p* = 0.31). 76.7% of the participants were visiting the higher grammar school (German “Gymnasium”) and 23.3% the lower grammar school (German “Mittelschule”). Parental education ranged from no school education (7) to doctoral degree (1) with an average education of *M* = 3.38 (*SD* = 1.45), representing a university diploma. For further details about the sample see Table 2. A urine test assured no use of illicit drugs (e.g. cannabis, heroin, cocaine) at the day of assessment.

**Table 2:** Participant characteristics (n = 104).

Age in years at T1	M = 14.52, SD = 0.32, range 13.83–14.99
Age in years at T2	M = 16.55, SD = 0.34, range 15.86–17.21
Interscan interval in years	M = 2.03, SD = 0.11, range 1.84–2.38
No. of females	N = 54 (51.9%)
No. of right-handers	93 (1 bimanual, 10 left)
IQ at T1^a^	M = 114, SD = 10, range 86–135
IQ at T2^b^	M = 115, SD = 11, range 91–145
Pubertal status^c^ at T1	M = 3.65, SD = 0.65, i.e. mid- to late pubertal status
Pubertal status at T2	M = 4.18, SD = 0.57, i.e. late pubertal status

*Note*. ^a^measured with the Wechsler Intelligence Scale For Children (WISC) that consisted of the subtests Similarities, Block Design, Vocabulary, and Matrices^61^; ^b^measured with the Wechsler Adult Intelligence Scale (WAIS) that consisted of the same subtests as WISC and additionally the Letter-Number Sequencing, Symbol Search, Digit Span, and Coding^62^; ^c^Pubertal status ranges from 1 for prepubertal to 5 for postpubertal status, measured with the Pubertal Development Scale (PDS^63^).

### Paradigms

For an overview of the main characteristics of the three paradigms see Table 3. In the emotional attention task, participants had to decide whether a pair of visual target stimuli was identical or not while another pair was presented as a distractor. Participants were not asked to attend to a particular emotional category but cued spatially by an arrow pointing in the direction of the two stimuli. Each trial consisted of a pair of pictures from one of three emotional categories (positive, neutral, negative) and a pair of non-emotional pictures. The emotional pictures were taken from the International Affective Picture System (IAPS^58^); and the non-emotional pictures were created by shredding the chosen IAPS pictures with GIMP (www.gimp.org). For further details see Vetter *et al*.^16^ and Pilhatsch *et al*.^15^ and Supplement S2.

**Table 3:** Overview of task characteristics.

	emotional attention	cognitive control	intertemporal choice
No. of trials of the chosen contrast/total task trials	20/120	64/256	90/90
Duration in min	15	21	25
Regressors of interest	negative attended > implicit baseline	switch incongruent > implicit baseline	intertemporal decision phase > implicit baseline
Task design	event-related	event-related	event-related
Regions of interest			
Task-based	mPCF	dACC	ACC
	IFG	dlPFC	Par-Sup
	Amy	PPC	VS
	FG		FG
Developmental	IFG	none	none
	ACC		
control region	Sup-Occ	Sup-Occ	Sup-Occ

*Note*. mPFC – medial prefrontal cortex, IFG – inferior frontal gyrus, Amy – Amygdala, FG – fusiform gyrus, ACC – anterior cingulate cortex, Sup-Occ – superior occipital lobe, dACC – dorsal anterior cingulate cortex, dlPFC – dorsolateral prefrontal cortex, PPC – posterior parietal cortex, Par-Sup – superior parietal lobe, VS – ventral striatum.

The first screen of the cognitive control task was an arrow consisting of two triangles pointing in one (left, right, up or down) direction and a red dot located either at the tip or the tail of the arrow. Participants were instructed to move a joystick in the direction indicated by the arrow or the dot. The shape of the background served as a task cue: If the background was rectangular, participants had to move the joystick in the direction of the arrow and ignore the position of the dot; conversely, if the background was circular, participants had to respond to the position of the dot while ignoring the arrow direction. Stimuli could be congruent, i.e. dot and arrow were pointing in the same direction, or incongruent, i.e. the dot and the arrow were pointing in opposite directions. For further details see Mennigen *et al*.^17^, Rodehacke *et al*.^18^.

In the intertemporal choice task participants had to choose between a larger later reward, which changed from trial to trial and a fixed immediate reward, which was instructed beforehand but not shown during scanning. In the current paper, the contrast of interest was the phase of the presentation of the potential later reward, i.e. the intertemporal decision phase, which refers to the process of comparing both alternatives in a given trial (fixed immediate or later reward). The task started with a behavioral training session to estimate the individual impulsivity parameter *k*, which was used to adapt the scanning paradigm to the subjects’ impulsivity. For more details see Ripke *et al*.^22^ and Ripke *et al*.^56^.

### Task presentation and order

The paradigms were presented with a LCD-based display system which was mounted on the head-coil (NordicNeuroLab AS, Bergen, Norway). Behavioral data were collected with a joystick (Resonance Technology Inc., Northridge, CA, USA) for the cognitive control task and by ResponseGrips (©NordicNeuroLab) with a button on a grip in each hand for the emotional attention and intertemporal choice task. Task presentation and recording of the behavioral responses was performed using Presentation® software (version 11.1, Neurobehavioral Systems, Inc., Albany, CA). Each task was preceded by a practice session. Since the tasks were assessed within an overall project including a large behavioral and fMRI battery, the order of tasks varied slightly between time points. At age 14, the order of paradigms was emotional attention, cognitive control and intertemporal choice on three different days within two weeks. At age 16 first the cognitive control and then the intertemporal choice task were assessed on the same day followed by the assessment of the emotional attention task within two weeks.

### Functional imaging

#### Image acquisition

For all three paradigms and across both sessions, image acquisition remained the same. MRI data was acquired using a 3 T whole-body MR tomograph (Magnetom TRIO, Siemens, Erlangen, Germany) with a 12-channel head coil. For all paradigms and across both sessions, an identical standard Echo Planar Imaging (EPI) sequence was used for functional imaging (TR/TE: 2410/25 ms; flip angle: 80°). FMRI scans were obtained from 42 transversal slices. Voxel size was 3 × 3 × 3 mm (slice thickness: 2 mm with 1 mm gap; FOV: 192 × 192 mm; in-plane resolution 64 × 64 pixels). Furthermore, a 3D T1-weighted magnetization-prepared rapid gradient echo (MPRAGE) image data set was acquired (TR/TE: 1900/2.26 ms; FOV: 256 × 256 mm; 176 slices; 1 × 1 × 1 mm voxel size; flip angle: 9°). Scanning settings and protocols were identical for all three paradigms and across both time points.

### Analysis of fMRI data

FMRI data analyses were performed using SPM5 (Wellcome Trust Center of Neuroimaging, London, UK) and were the same for both time points per paradigm.

#### Preprocessing

For preprocessing, which was identical for all three tasks, functional images were first slice-time corrected by using the middle slice as reference and realigned to the first image (by 6° rigid spatial transformation). Afterwards they were spatially normalized into Montreal Neurological Institute (MNI) space and spatially smoothed with an 8 mm full-width half maximum Gaussian kernel.

#### Statistical analysis

For all paradigms first-level contrasts were computed with a fixed effects analysis for each participant based on the general linear model by modeling the different conditions as regressors of interest within each voxel for the whole brain. For each paradigm, the six subject-specific movement regressors, which were derived from the rigid-body realignment, were included as covariates of no interest. A high-pass filter with cut-off 128 s was applied to remove the low frequency physiological noise^59^ for each paradigm. Also an autoregression, AR(1), model was employed for the residual temporal autocorrelation^59^ for each paradigm. Contrasts of interest (see Table 3) were computed for each paradigm within each subject. The first-level contrast images from the weighted beta-images were used for second-level whole brain random-effects analyses to allow for population inference. For a detailed description of the first- and second-level analyses for each paradigm see S3 in the supplement.

### Definition of ROIs

**Figure 4:**
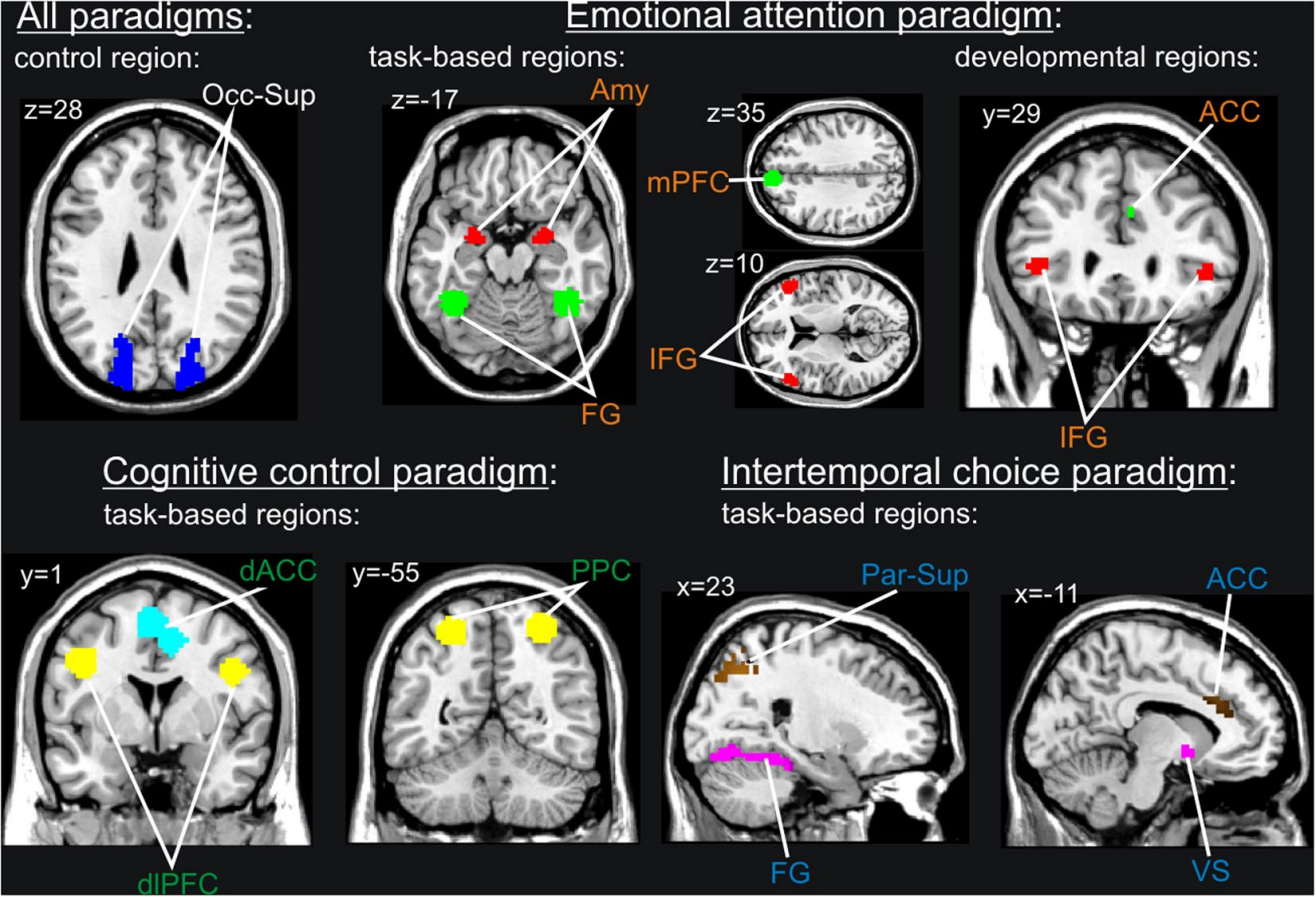
Regions of interest that were used to calculate ICC for all paradigms. The control regions for all paradigms were the left and right superior occipital lobe.

For an overview of used ROIs see Fig. 4. ROIs were defined based on a priori hypotheses regarding activation in the respective tasks and based on functional masks resulting from the whole-brain analyses of each task at the first time point, i.e. age 14^16, 17, 22^. 10 mm spheres were placed around the peak coordinates (see Table S3 in the Supplementary Materials) of the whole brain analyses at age 14 and thus final ROIs created. Additionally, bilateral superior occipital ROIs using the WFU-PickAtlas with the Automated Anatomical Labeling Atlas (AAL) were created that served as control regions for all three tasks. Specific ROI approaches for each paradigm are described in the following.

#### Emotional attention paradigm

For this paradigm, we focused on attending negative versus attending neutral stimuli for functional ROI extraction for two reasons: The attending negative in contrast to the attending neutral condition resulted in slower reaction times which indicates an attentional capture effect^16^. Second, separate ROIs for emotional attention could be created by subtracting the neutral contrast (but not by subtracting the implicit baseline since almost the whole brain was activated). The amygdala was chosen as an additional ROI because it was also activated for negative target stimuli in the paradigm but defined the whole amygdala as a larger cluster anatomically using the WFU-PickAtlas with the Talairach Daemon (TD) Brodman atlas (following^15, 16^). Furthermore, for this paradigm, two ROIs with developmental effects were analyzed that emerged from higher activation during presentation of emotional target and distractor stimuli for age 16 versus 14 in the right and left inferior frontal gyrus (IFG) and the ACC^16^, see Table S3 in the Supplementary Materials.

#### Cognitive control paradigm

ROIs were created based on a conjunction analysis^17^. Switch- and incongruence-related activity overlapped in bilateral dACC, dlPFC and PPC. We chose trials with co-occurrence of incongruence and switch (switch incongruent trials > implicit baseline) because of two reasons. These trials led to a steep increase in reaction time and error rate therefore reflecting a high level of cognitive control^17^. Further, task switch and incongruence trials robustly and independently activated the core regions of the cognitive control network^17^.

#### Intertemporal choice paradigm

For this paradigm, ROIs of the fusiform gyrus, the superior parietal lobe as well as the ACC were created by using the overlap of functional activation of the intertemporal decision phase^22, 56^ and anatomical regions using the WFU-PickAtlas with the AAL atlas. The overlap with anatomical regions was necessary to create distinct ROIs because the activation spanned one very large cluster across the whole brain. We additionally chose the VS as a ROI since it is highly relevant for reward paradigms. The anatomical ROIs of the VS were created with the WFU-PickAtlas using the AAL atlas.

### Analyses of reliability

#### Behavioral reliability

Behavioral ICCs_(3,1)_ were calculated using SPSS v21 (IBM Corp., Armonk, USA). For the emotional attention and the cognitive control paradigm, reaction times of the specific conditions and overall reaction times across conditions and for the intertemporal choice paradigm, log-transformed discount parameters were analyzed for reliability.

#### FMRI reliability

FMRI ICCs were calculated with the ICC toolbox of Caceres *et al*.^60^. We used the intra-voxel reliability “*ICC*_*v*_ *”* obtained by using the contrast value of each voxel within each ROI of each individual subject. The population estimate was obtained by bootstrapping with 1,000 re-samples of participants, of which medians and standard errors are reported. Additionally, whole brain ICCs were calculated, since this is the strictest criterion and potentially the most valuable reliability measure as it yields a global measurement of test-retest agreement^2^. ICCs were classified according to Cicchetti^4^ as poor, <0.40, fair, 0.41–0.60, good, 0.61–0.75, and excellent, >0.75 (see also ref. 5).

## Electronic supplementary material

Supplementary Material
