# Supplementary material for: Reliability in adolescent fMRI within two years – a comparison of three tasks
Source: Sci Rep. 2017 May 23;7:2287. doi: 10.1038/s41598-017-02334-7 (PMC5442096; doi:10.1038/s41598-017-02334-7)
Supplement: Supplementary file 1 — Supplementary Material [file 41598_2017_2334_MOESM1_ESM.doc]

# Reliability in adolescent fMRI within two years – a comparison of three tasks

Nora C. Vetter^1,2,3,*^, Julius Steding ^1,2,4^, Sarah Jurk^1^, Stephan Ripke^1^, Eva Mennigen^1^, Michael N. Smolka^1,*^

^1^ Department of Psychiatry and Neuroimaging Center, Technische Universität Dresden, Germany

^2^ Department of Child and Adolescent Psychiatry, Faculty of Medicine of the TU Dresden, Germany

^3^Department of Psychology, Bergische Universität Wuppertal, Germany

^4^Division of Psychological and Social Medicine and Developmental Neurosciences, Faculty of Medicine of the TU Dresden, Germany

Corresponding authors:

Nora C. Vetter / Michael N. Smolka

Technische Universität Dresden

Faculty of Medicine Carl Gustav Carus

Department of Psychiatry and Psychotherapy

Section of Systems Neuroscience

01187 Dresden, Germany

E-Mail: nora.vetter@tu-dresden.de, michael.smolka@tu-dresden.de

**Supplementary Material**

*S1:* *Detailed**exclusion criteria of all three paradigms:*

The overlapping sample of the following three sub-samples of the paradigms was built:

| Emotional  Attention: | 187 participants were successfully assessed at both time points of which 144 (73 female) were included in the analysis of two time points. The others were excluded because of 1) excessive head movements (N=23), 2) low behavioral performance during the fMRI task, i.e. more than 25% incorrect answers (N=14), or mean reaction times higher than 3 standard deviations from the mean of their age samples at age 14 or 16 (N=6). |
| --- | --- |
| Cognitive  control: | 200 participants were successfully assessed at both time points of which 144 (73 female) were included in the analysis of two time points. Reasons for exclusion were the following: 1) ADHD (N=3), 2) excessive head movements (N=17), 3) failed normalization (N=14), 4) technical problems during scanning (N=12), and 5) low behavioral performance during the fMRI task, i.e. one of the four conditions contained less than 50 % of correct trials (N=10). |
| Intertemporal choice: | 192 participants were successfully assessed at both time points of which 174 (84 female) were included in the analysis of two time points. Reasons for exclusion were the following: 1) any DAWBA diagnosis (N=4), 2) technical problems during scanning (N=8), 3) low behavioral performance during the fMRI scanning session, i.e. only few responses (N=6). |

*S2: Emotional attention paradigm, example trials (from Vetter et al., 2015)*


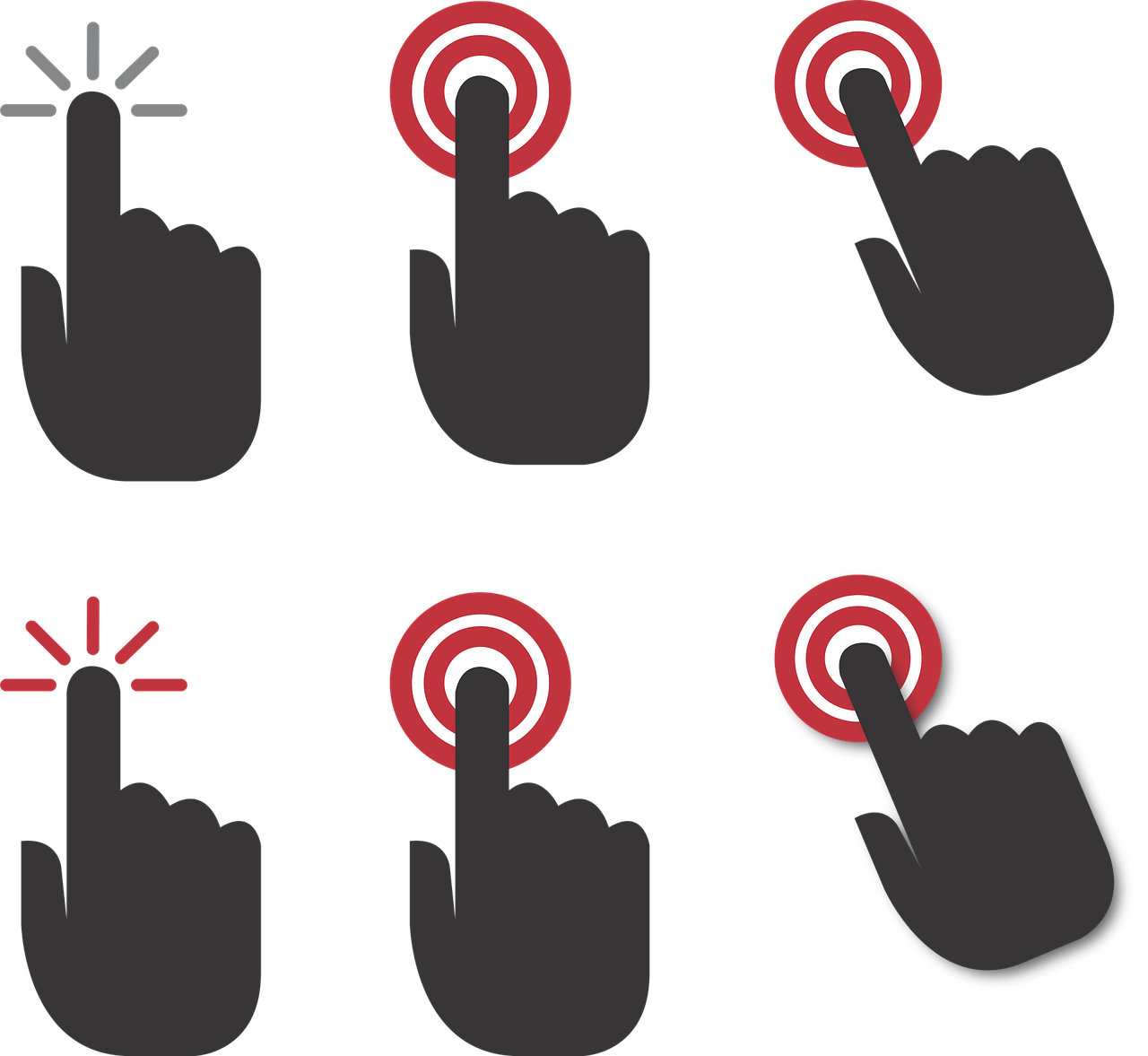

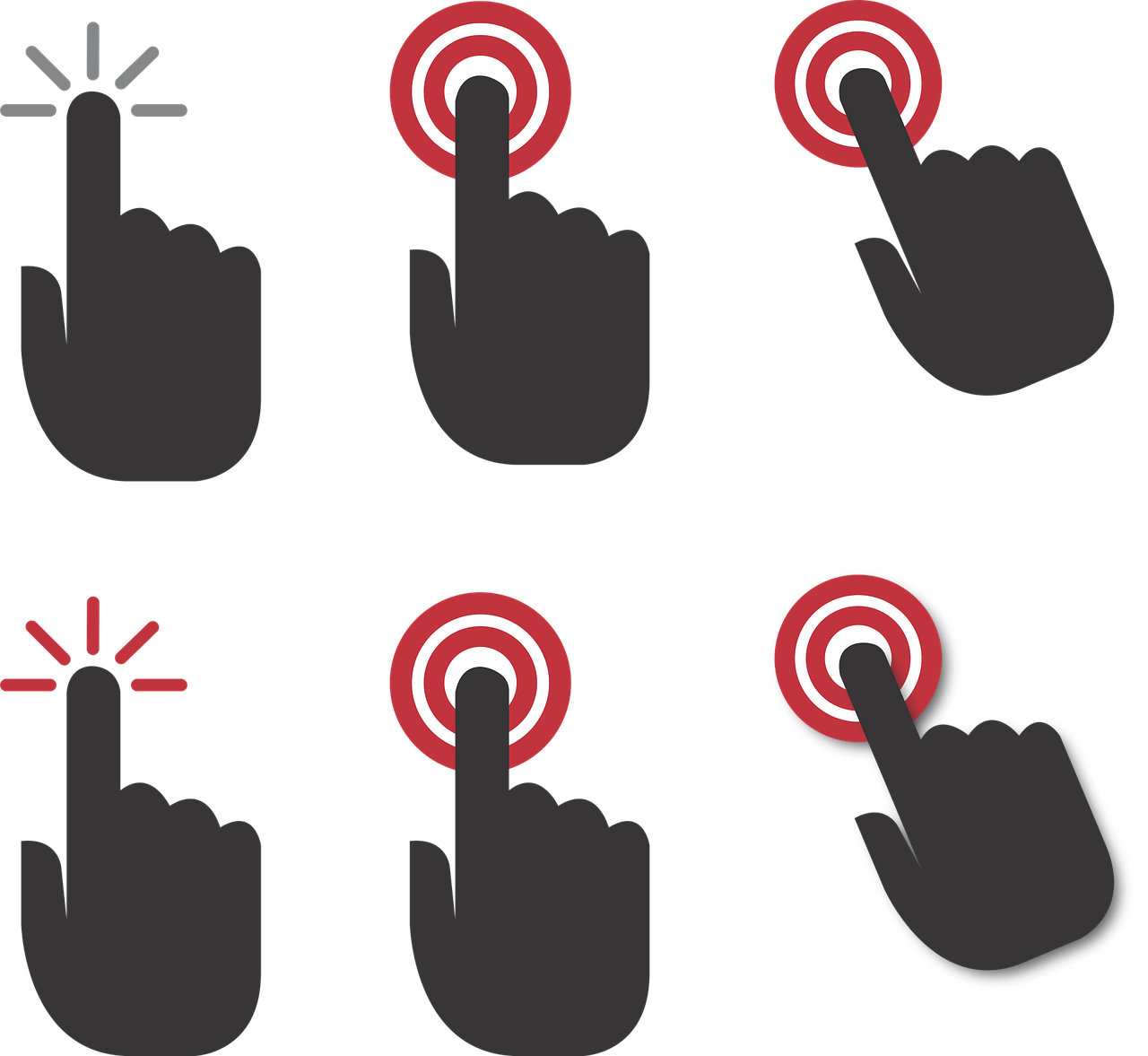

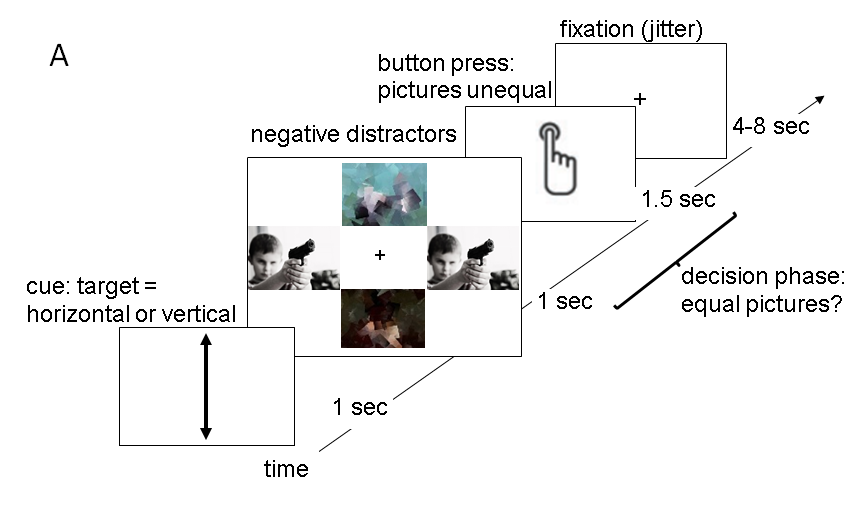

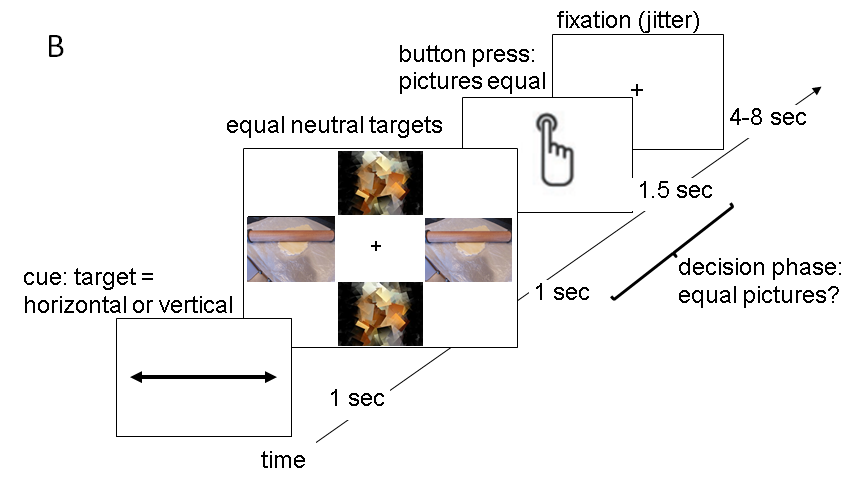


a) Example trial for the Ignoring emotion condition. b) Example trial for the Attending emotion condition.

S3: First and second-level statistics for each paradigm underlying ROI creation

*Emotional attention (approach from Vetter et al., 2015)*

First level: Six regressors of interest, attending (1) negative, (2) positive, and (3) neutral emotion as well as ignoring (4) negative, (5) positive, and (6) neutral emotion, were modeled at the point of presentation as stick functions convolved with a canonical hemodynamic response function. Additionally, trials with missing or wrong responses were modeled as a separate regressor, i.e. only correct answers were analyzed.

Second level: An ANOVA was computed using a 2 x 2 x 2 full factorial model with the within-subject factors age (age 14, age 16), attention (attending emotion, ignoring emotion), and emotional valence (negative versus neutral, positive versus neutral) using contrasts 1, 2, 3, and 4. On a whole brain level the resulting set of signiﬁcant voxel values constituted an SPM map.

For non-developmental ROIs, a statistical threshold of *p* ≤ .001 uncorrected at the voxel-level and *p_FWE_* < .05 at the cluster level was employed.

For developmental ROIs, a statistical threshold of *p* ≤ .05 (corrected for multiple tests on the cluster threshold criterion) equivalent to a minimum cluster size of 32 voxels was employed with a voxel-level threshold of *p* ≤ .001.

*Cognitive control (approach from Mennigen et al., 2014)*

First-level: Regressors were built from the 16 different conditions resulting from the 2 (task) x 2 (task transition) x 2 (previous trial congruence) x 2 (present trial congruence) design and modeled at the point of presentation as stick functions convolved with a canonical hemodynamic response function. All error and missing trials (trials in which participants did not react within 2.1 seconds) as well as correct trials following error and missing trials (post-error/post-missing trials) were considered as additional regressors. For each subject, these contrast images (16 conditions, error, post-error and “omitted” contrasts) were compiled. For further second level random effects analyses we did not include the factors previous trial congruence and task in our model, since we were only interested in the effects of task transition and incongruence. Thus, we ran a 2*2 full-factorial design at the group level with the factors task transition and response incongruence including the respective 4 single subject contrast images (i.e., repeat congruent, repeat incongruent, switch congruent, switch incongruent, all versus implicit baseline).

Second-level: For group statistics we ran a 2 x 2 full-factorial design with the factors task transition (repeat vs. switch) and response incongruence (congruent vs. incongruent) for group level analyses. We applied a whole brain analyses and used a threshold of *p* < 0.05 (FDR-corrected) with a cluster size of at least 25 contiguous voxels. Further, a conjunction analysis was conducted to analyze the overlap between the switch vs. repeat and the incongruent vs. congruent effect with a cluster size of at least 20 contiguous voxels.

*Intertemporal choice (approach from Ripke et al. 2012)*

First-Level: The first-level model consisted of four regressors for different events of valid trials. For these trials, the intertemporal decision phase (presentation of the delayed reward) and the subsequent motor response/ feedback (separated for responses with the left and right hand) were modelled as events using the canonical hemodynamic response function. The subjective reward value (computed with the subjective impulsivity parameter, *k*, from the initial training session of the experiment) of each delayed reward presented was additionally included as parametric modulation of the respective event. The presentation of the delayed reward of invalid trials was modelled as a further regressor. For trials with implausible choices, the subsequent motor response was modelled using the same regressor as invalid trials.

Second-Level: Main effects regarding brain activity related to the intertemporal decision phase were analyzed on a whole brain basis and the statistical threshold was set to T=4.35 (*p* < 10^-5^).

| **Table S3.** Details on the functional regions of interest: peak voxels from the second-level contrasts in MNI reference space. | | | | | | |
| --- | --- | --- | --- | --- | --- | --- |
| Paradigm | Contrast | Region | R/L | x, y, z | k | t-value |
| Emotional attention | Negative > neutral attended | FG | R | 42, -48, -18 | 914 | 8.78 |
|  |  |  | L | -39, -45, -18 | 733 | 8.6 |
|  |  | IFG (part of an extending cluster) | R | 48, 24, 15 | * |  |
|  |  |  | L | -48, 24, 15 | 246 | 4.31 |
|  |  | mPFC | L | -6, 51, 33 | 193 | 5.18 |
|  | Developmental: Age 16 > age 14 (all conditions) | IFG | R | 45, 33, 0 | 46 | 4.52 (z-value) |
|  |  |  | L | -42, 30, 3 | 34 | 4.52 (z-value) |
|  |  | ACC | R | 3, 21, 27 | 42 | 3.95 (z-value) |
| Cognitive control | Conjunction analysis (switch > repeat ∩ incongruent > congruent) | dACC | R | 9, 6, 48 | 882 | 3.52 |
|  |  |  | L | -3, -3, 57 |  | 4.18 |
|  |  | dlPFC | R | 45, 6, 30 | 22 | 3.46 |
|  |  |  | L | -42, -3, 36 | 89 | 3.74 |
|  |  | PPC | R | 30, -54, 60 | 1270 | 5.00 |
|  |  |  | L | -24, -57, 57 | 1268 | 6.40 |
| Intertemporal choice | Intertemporal decision phase > implicit baseline | ACC, Par-Sup, FG as part of a large cluster | L | -12, -96, -6 | 26,315 | 21.88 |
| *Note*. R – right hemisphere; L – left hemisphere; *although the right IFG was not activated it was added with the same coordinates as the left IFG at the right side to compare activity in the developmental right IFG. | | | | | | |

*S4:* *Exploratory analyses: Relationship of behavioral data and functional reliability:*

We additionally explored the correlation between the ICC_v_ and the reaction times for the emotional and the cognitive paradigm and the log-transformed discount parameter for the reward paradigm with correction for multiple comparisons. No significant correlation was found.
